# Reliability of radiographic measurements for acute distal radius fractures

**DOI:** 10.1186/s12880-016-0147-7

**Published:** 2016-07-22

**Authors:** Narelle J. Watson, Saeed Asadollahi, Frank Parrish, Jacqueline Ridgway, Phong Tran, Jennifer L. Keating

**Affiliations:** Allied Health Department & School of Primary Health Care, Western Health & Monash University, Melbourne, Victoria Australia; Nepean hospital, Penrith, NSW Australia; Peninsula Health, Melbourne, Victoria Australia; I-TeleRAD, Melbourne, Victoria Australia; Orthopaedic Department Western Health, Melbourne, Victoria Australia; Department of Physiotherapy, School of Primary Health Care, Faculty Medicine Nursing and Health Sciences, Monash University, Melbourne, Victoria Australia

**Keywords:** Distal radius fracture, Radiographs, Reliability

## Abstract

**Background:**

The management of distal radial fractures is guided by the interpretation of radiographic findings. The aim of this investigation was to determine the intra- and inter-observer reliability of eight traditionally reported anatomic radiographic parameters in adults with an acute distal radius fracture.

**Methods:**

Five observers participated. All were routinely involved in making treatment decisions based on distal radius fracture radiographs. Observers performed independent repeated measurements on 30 radiographs for eight anatomical parameters: dorsal shift (mm), intra-articular gap (mm), intra-articular step (mm), palmar tilt (degrees), radial angle (degrees), radial height (mm), radial shift (mm), ulnar variance (mm). Intraclass correlation coefficients (ICCs) and the magnitude of retest errors were calculated.

**Results:**

Measurement reliability was summarised as high (ICC > 0.80), moderate (0.60–0.80) or low (<0.60). Intra-observer reliability was high for dorsal shift and palmar tilt; moderate for radial angle, radial height, ulnar variance and radial shift; and low for intra-articular gap and step. Inter-observer reliability was high for palmar tilt; moderate for dorsal shift, ulnar variance, radial angle and radial height; and low for radial shift, intra-articular gap and step. Error magnitude (95 % confidence interval) was within 1–2 mm for intra-articular gap and step, 2–4 mm for ulnar variance, 4–6 mm for radial shift, dorsal shift and radial height, and 6–8° for radial angle and palmar tilt.

**Conclusions:**

Based on previous reports of critical values for palmar tilt, ulnar variance and radial angle, error margins appear small enough for measurements to be useful in guiding treatment decisions. Our findings indicate that clinicians cannot reliably measure values ≤1 mm for intra-articular gap and step when interpreting radiographic parameters using the standardised methods investigated in this study. As a guide for treatment selection, palmar tilt, ulnar variance and radial angle measurements may be useful, but intra-articular gap and step appear unreliable.

**Electronic supplementary material:**

The online version of this article (doi:10.1186/s12880-016-0147-7) contains supplementary material, which is available to authorized users.

## Background

Comprising 2.5 % of all Emergency Department (ED) presentations, fracture of the distal radius is the most common skeletal fracture type [1]. It occurs in approximately 10 % of Caucasian women over 65 years [2]. After cast removal, immediate functional limitations include loss of strength (particularly grip) and range of movement [3, 4]. A decade following fracture, ongoing pain and reduced function of the wrist and hand can still occur with heavy tasks [5].

Radiographs of the distal radius are used for diagnosis, to guide treatment choices, assess fracture reduction and monitor healing. There are no standardised, evidence-based methods for interpreting radiographic parameters. Eight anatomic parameters of distal radius fracture have been described. These are dorsal shift (mm), intra-articular gap (mm), intra-articular step (mm), palmar tilt (degrees), radial angle (degrees), radial height (mm), radial shift (mm), and ulnar variance (mm) [6]. Relationships have been described between functional outcome and the anatomical parameters of intra-articular gap and step [7–9], dorsal [10–12] and palmar tilt [13], radial angle [14, 15], radial height [10, 16, 17], radial shift [18] and ulnar variance [19, 20]. No such relationships have been described for dorsal shift suggesting it may have no clinical utility, may not be adequately reliable and/or its close correlation with dorsal tilt [21] renders this measurement less important. This investigation explored the reliability of these eight parameters in preparation for a study of their utility in guiding treatment decisions.

We assessed the error associated with radiographic interpretation of acute distal radius fractures and whether the errors associated with measurements were small enough for measurements to be used confidently in fracture management. We investigated the intra- and inter-observer reliability of eight anatomic parameters in skeletally mature patients with an acute distal radius fracture using digitised radiographs. This investigation extends and updates the work of Kreder et al. [6] by using a larger sample of radiographs (30 acute fractures) and the computerised images and measurement procedures used in current practice. The majority of choices regarding treatment for distal radius fractures occur in the acute period. This investigation utilized radiographs of acute distal radius fractures in contrast to the healed distal fracture radiographs utilized by Kreder et al. [6].

## Methods

### Participants: selection of radiographs

Posteroanterior (PA) and lateral wrist radiographs of all patients with distal radius fractures presenting to a large outer metropolitan ED in Victoria, Australia during the period July 2009 to January 2010 were retrospectively selected for review. Standardised positioning of neutral forearm rotation was adopted for the PA and lateral views. Inclusion criteria for radiographs were skeletal maturity, fracture within 3 cm of the distal end of the radius, and presenting to the ED within seven days of fracture. Exclusion criteria were pathological fracture and evidence of previous distal radius fracture on the affected side. Radiographs meeting the inclusion/exclusion criteria were assembled and stratified by fracture deformity to either Group A (mild deformity) or Group B (severe deformity) based on decision rules defining estimates of severity (Table 1). In the absence of guidelines, decisions regarding cut points separating mild from severe were arbitrary and intended only to enable the spectrum of injury to be appropriately represented in the assembled targets. Fifteen radiographs from each group were randomly selected based on a computer generated sequence. The sample size of 30 repeated measurements for each observer was chosen as difference scores for repeated measures in samples of 30 or more are likely to assume a normal distribution [22].

**Table 1:** Radiographic characteristics for classification as mild or severe deformity

Group A Mild deformity: must meet all criteria	Group B Severe deformity: must have at least one criteria
Intra-articular step: ≤ 2 mm	Intra-articular step: > 2 mm
Intra-articular gap: ≤ 2 mm	Intra-articular gap: > 2 mm
Dorsal tilt: ≤ 10°	Dorsal tilt: > 10°
Volar tilt: ≤ 20°	Volar tilt: > 20°

### Participants: selection of assessors

To accommodate the influence of site specific practices, observers were invited from two independent health networks. Health professionals were invited to participate if their role included making treatment decisions based on radiographic images of upper limb fractures. Invitations were sent to ED, orthopaedic and radiology staff, and upper limb specialists. Potentially eligible professional groups included orthopaedic consultants and registrars, radiologists, ED consultants and registrars, and advanced practice musculoskeletal physiotherapists. A total of 10 observers (five from each health network) meeting these criteria were invited to participate. Ensuring at least two observers from each health network, the first five observers to consent were enrolled in the investigation.

### Observer training

**Fig. 1:**
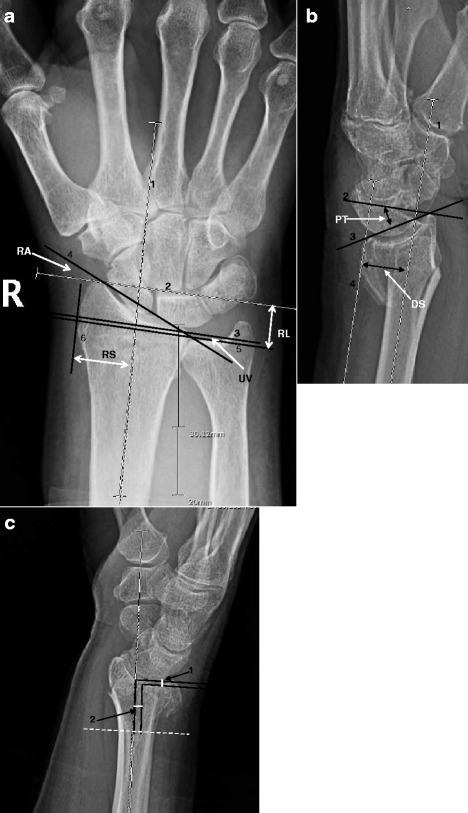
**a** Posterioanterior measurement guidelines as described in Kreder et al. [6]. **b** Lateral measurement guidelines as described in Kreder et al. [6]. **c** Step and gap measurement as described in Kreder et al. [6]. **a** RA, radial angle; RL, radial length; UV, ulnar variance; RS, radial shift. 1) This line represents the long axis of the radius. The center of the radius shaft is determined at 3 cm and 5 cm below the mid-region of the proximal lunate articular surface. 2) A line perpendicular to the center long axis of the radius is drawn at the level of the most distal aspect of the radial articular surface. 3) A line perpendicular to the central long axis of the radius is drawn at the level of the ulnar margin of the distal radial articular surface. 4) The radial and ulnar margins of the distal radial articular surface are connected. 5) A line perpendicular to the central long axis of the radius is drawn at the level of the distal ulnar articular surface. 6) A line tangential to the most radial point on the radial metaphysis is drawn parallel to the central long axis of the radius. **b** PT palmar tilt angle (dorsal tilt=negative palmar tilt); DS, dorsal shift. 1) This line represents the long axis of the radius. The center of the radial shaft is determined at 3 cm and 5 cm below the mid-region of the proximal lunate articular surface. 2) A line perpendicular to the central long axis of the radius is drawn at a convenient level. 3) The dorsal and anterior margins of the distal radial articular surface are connected. 4) A line tangential to the most dorsal point on the radial metaphysis is drawn parallel to the central long axis of the radius. **c** 1) Step-off at the distal radius articular cortical margin is measured by drawing lines perpendicular with the central long axis of the radius from the most distal margin of each side of the cortical discontinuity. 2) Gap deformity is measured by dropping lines that are parallel from the central long axis of the radius from the most distal margin of each side of the cortical deformity. The gap distance is measured along a line perpendicular to the central long axis of the radius.

Prior to commencing this study, each observer completed a self-directed tutorial that provided standardised instructions and examples illustrating measurement techniques for assessing each of the eight parameters. Observers were then given three wrist radiographs of acute distal radius fractures and asked to measure each of the eight anatomical parameters. The standardised method developed by Kreder et al. [6] for measuring these eight anatomic parameters at the distal radius was followed (Fig. 1a, b, c) [6]. Observers utilised picture archiving and communication system (PACS) computerised images through the Synapse [23] display system that includes measurement calibration. Two views, PA and lateral, were utilised to obtain measurements for each of the eight parameters. Observers were asked to save all working lines used in computations. Each observer was then provided with written and verbal feedback from the principal investigator on departures from standardised measurement techniques. Participants were asked to repeat measurements where incorrect technique was noted and again save measurement decision rules for review and feedback from the principal investigator. Observer training was completed over a two week period. The purpose of this preparation was to identify and minimise sources of systematic and random error in reading images.

### Measurement parameters

The five observers were asked to measure the following parameters for each of the 30 radiographs: dorsal tilt (degrees), intra-articular gap (mm), intra-articular step (mm), palmar tilt (degrees), radial angle (degrees), radial height (mm), radial shift (mm), and ulnar variance (mm) using methods described in the observer training tutorials.

### Measurements

On the first measurement occasion, observers were presented, in random sequence, with 30 fully de-identified radiographs with no unique identifying features. No earlier than two weeks and no later than three weeks later, observers were presented with the same set of radiographs, again in random sequence and without access to measurements taken on the first measurement occasion. To reduce the potential impact of measurement fatigue, observers were instructed to disperse their measurements over a two week period.

### Statistics

Data were analysed using the recommendations by Rankin and Stokes [24] (correlational indices) and Bland and Altman [25] (metricated error estimates). The ICC quantifies the relationship between two variables with *r* = 1 indicating perfect agreement and *r* = 0 indicating no agreement.

#### Intra-observer reliability

Intra-observer ICCs were calculated for each of the eight anatomical parameters on data produced from a two-way repeated analysis of variance (ANOVA). The test retest values for each observer were compared for each anatomical parameter. Using equations provided by Fleiss [26] for repeated measurements by the same observer, the ICC(1, 1) was calculated using the formula:

1 $$ \mathrm{I}\mathrm{C}\mathrm{C}\left(1,1\right)=\mathrm{B}\mathrm{M}\mathrm{S}\hbox{-} \mathrm{W}\mathrm{M}\mathrm{S}/\mathrm{B}\mathrm{M}\mathrm{S}+\left(\mathrm{k}\hbox{-} 1\right)\mathrm{W}\mathrm{M}\mathrm{S} $$

where *k* is the number of measurements, and mean squares (MS) of variance estimates were obtained from ANOVA: *BMS* (between-subjects variance) and *WMS* (within subjects variance).

Bland and Altman [25] analysis was used to quantify agreement between measurements made by the same observer; the difference between two measurements was plotted against the average of the two measurements. The 95 % confidence intervals (CIs) around the mean differences were calculated using the standard errors in estimates of the mean and a t multiplier appropriate for the sample size (2.05) [25].

#### Inter-observer reliability

The first set of measurements of the eight anatomical parameters for each of the 30 radiographs were used to calculate the inter-observer reliability. The analyses were repeated for fractures dichotomised by severity of deformity to assess whether reliability changed with fracture severity. Variance estimates were derived from a one-way repeated ANOVA. The ICC(3,1) was calculated based on recommendations and equations by Shrout and Fleiss [27] for inter-observer reliability:

2 $$ \mathrm{I}\mathrm{C}\mathrm{C}\left(3,1\right)=\mathrm{B}\mathrm{M}\mathrm{S}\hbox{-} \mathrm{E}\mathrm{M}\mathrm{S}/\mathrm{B}\mathrm{M}\mathrm{S}+\left(\mathrm{k}\hbox{-} 1\right)\mathrm{E}\mathrm{M}\mathrm{S} $$

The ICC(3,1) was chosen as each radiograph in the current investigation was rated by each of the same *k* observers who were the only observers of interest. As the observers for this investigation were selected from the general population, ICC(1,1) could have been used, however the more conservative equation was chosen. Bland and Altman [25] analysis was again applied to quantify inter-observer agreement.

As this investigation involved exploring agreement between more than two fixed observers, a representative average of the reliability between pairs within the five observers was calculated using an overall concordance correlation coefficient (OCCC) based on recommendations and equations by Barnhart et al. [28]. The OCCC provides an overall correlation that takes into account the correlation between individual pairs of observers.

A number of investigations have recommended conservative management for distal radius fractures when intra-articular gap or step is less than 1 mm [7–9]. Intra-articular gap and step measurements were therefore converted to a dichotomy of less than or equal to 1 mm or greater than 1 mm and pairwise inter-observer agreement values (kappa) were calculated. Further pairwise inter-observer agreement values (kappa) were calculated when intra-articular gap and step measurements were converted to a dichotomy of presence (any gap or step recorded) or absence (zero gap or zero step recorded) of intra-articular step or gap. Data from the first set of measurements was used for the conversions to dichotomies.

## Results

The professional roles of the five observers who reviewed radiographs were orthopaedic surgeon (upper limb), orthopaedic registrar, ED consultant, ED primary care advanced practice musculoskeletal physiotherapist and radiologist.

Measurement reliability was summarised as high (ICC > 0.80), moderate (0.60–0.80) or low (<0.60). Intra-observer reliability was high for dorsal shift and palmar tilt; moderate for radial angle, radial height, ulnar variance and radial shift; and low for intra-articular gap and step. Inter-observer reliability was high for palmar tilt; moderate for dorsal shift, ulnar variance, radial angle and radial height; and low for radial shift, intra-articular gap and step (Table 2). OCCC values ranged from 0.11 for intra-articular gap to 0.94 for palmar tilt (Table 2). ICC values appeared higher in the current investigation compared with Kreder et al. [6] for all parameters except radial shift and ulnar variance (Table 2). Error magnitude (95 % confidence interval) was within 1–2 mm for intra-articular gap and step, 2–4 mm for ulnar variance, 4–6 mm for radial shift, dorsal shift and radial height, and 6–8° for radial angle and palmar tilt (Table 3).

**Table 2:** Intra- and inter-observer ICCs & OCCCs for each anatomical parameter based on ANOVA output and Equations 1 and 2 are compared to data from Kreder et al. [6]

Anatomical parameter	Intra-observer	Inter-observer (using 1^st^ measurements)	OCCC^13^ (using 1^st^ measurements)	Kreder et al. [6] intra-observer	Kreder et al. [6] inter-observer
Dorsal shift	0.91	0.75	0.77	0.48	0.42
Intra-articular gap	0.56	0.30	0.11	0.37	0.35
Intra-articular step	0.54	0.31	N/A	0.22	0.27
Palmar tilt	0.89	0.93	0.94	0.71	0.74
Radial angle	0.80	0.66	0.66	0.39	0.38
Radial height	0.79	0.61	0.61	0.49	0.44
Radial shift	0.68	0.47	0.50	0.72	0.67
Ulnar variance	0.75	0.69	0.70	0.85	0.82

**Table 3:** Range of measurements, standard error of measurement (SEM) and 95 % confidence intervals (95 % CI) for each anatomical parameter using first set of measurements of 30 radiographs, compared to data from Kreder et al. [6] based on six radiographs

Parameter	Mean (SD)	Minimum, Maximum	SEM (inter-observer using 1^st^ measurements)	Upper limit 95 % CI	Minimum, Maximum Kreder et al. [6]
Dorsal shift (mm)	15.03 (4.85)	(3, 23.4)	2.42	4.97	(2, 19)
Intra-articular Gap (mm)	0.76 (1.13)	(0, 5.7)	0.94	1.94	(0, 5)
Intra-articular step (mm)	0.27 (0.62)	(0, 3.95)	0.52	1.06	(0, 4)
Palmar tilt (degrees)	−6.29 (14.28)	(−36, 42)	3.78	7.75	(−31, 24)
Radial angle (degrees)	18.18 (5.67)	(3, 30)	3.31	6.78	(3, 27)
Radial height (mm)	8.71 (4.07)	(0, 24)	2.54	5.21	(0, 14)
Radial shift (mm)	18.71 (3.00)	(13, 30.8)	2.18	4.48	(11, 25)
Ulnar variance (mm)	0.68 (1.96)	(−4.5, 5.7)	1.09	2.24	(−2,10)

Dichotomising intra-articular gap and step measurements as above or below 1 mm produced pairwise inter-observer agreement values ranging from −0.06 to 0.52 and −0.11 to 0.43 respectively (Tables 4 and 5). Dichotomising intra-articular gap and step measurements by the presence or absence of intra-articular involvement produced pairwise inter-observer agreement values ranging from −0.07 to 0.67 and 0 to 0.63 respectively (Tables 4 and 5).

**Table 4:** Kappa values for inter-observer agreement for intra-articular gap (taken from 1^st^ recording) using raw scores and scores dichotomised with recoding

Pairwise comparison	Uncoded data (as recorded)	Coding: 0 = < 1 mm 1 = equal or > 1 mm	Coding: 0 = unable to see gap 1 = able to see gap
P1P2	0.24	0.52	0.54
P1P3	0.16	0.39	0.53
P1P4	0.22	0.52	0.67
P1P5	0.03	0.08	0.06
P2P3	0.08	0.15	0.23
P2P4	0.27	0.25	0.60
P2P5	−0.03	−0.06	−0.07
P3P4	0.15	0.45	0.47
P3P5	0.02	0.09	0.05
P4P5	0.03	0.11	0.07

**Table 5:** Kappa values for inter-observer agreement for intra-articular step (taken from 1^st^ recording) using raw scores and scores dichotomised with recoding

Pairwise comparison	Uncoded data (as recorded)	Coding: 0 = < 1 mm 1 = equal or > 1 mm	Coding: 0 = unable to see step 1 = able to see step
P1P2	0.25	0.38	0.63
P1P3	0.13	0.07	0.29
P1P4	0.22	0.30	0.56
P1P5	0.00	0.00	0.00
P2P3	0.25	0.43	0.53
P2P4	0.23	0.14	0.56
P2P5	0.00	0.00	0.00
P3P4	0.11	−0.11	0.26
P3P5	0.00	0.00	0.00
P4P5	0.00	0.00	0.00

Dichotomizing data based on fracture severity resulted in a systematic improvement in inter-observer reliability values for more severe fractures with all measurements except dorsal shift (Table 6). No systematic reductions in error were seen for more severe fractures when calculations of standard error of measurement (inter-observer) were performed (Table 6).

**Table 6:** Inter-observer ICCs and SEMs (taken from 1^st^ recording) using data dichotomized for severity of fracture deformity

Anatomical parameter	ICC All 5 observers- 30 radiographs	ICC Grp 1 (mild deformity)- 15 radiographs	ICC Grp 2 (severe deformity)- 15 radiographs	SEM Grp 1 (mild deformity)- 15 radiographs	SEM Grp 2 (severe deformity)- 15 radiographs
Dorsal shift	0.75	0.76	0.71	3.09	1.46
Intra-articular gap	0.30	0.15	0.30	0.39	0.66
Intra-articular step	0.31	0.26	0.30	0.18	0.39
Palmar tilt	0.93	0.92	0.96	7.75	6.52
Radial angle	0.66	0.62	0.71	3.00	2.59
Radial height	0.61	0.49	0.74	2.07	1.95
Radial shift	0.47	0.27	0.55	1.18	1.77
Ulnar variance	0.69	0.67	0.70	0.93	1.00

Stratifying data based on professional subgroups e.g. isolating the analysis to the orthopaedic surgeon and radiologist, did not result in systematically higher reliability values. The highest correlations were obtained when data for all five observers were included in analysis.

### Bland and Altman

**Fig. 2:**
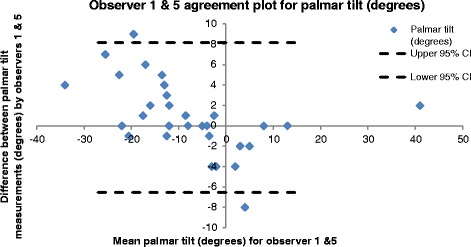
Bland and Altman [20] distribution plot for palmar tilt, showing the difference between measurements by observers 1 & 5 plotted against the average measurement for the two observers using data from the first set of measurements

**Fig. 3:**
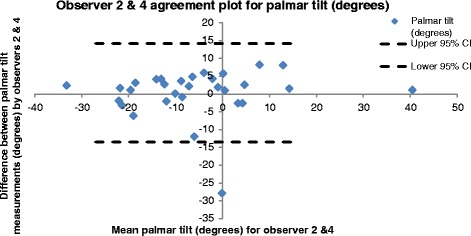
Bland and Altman [20] distribution plot showing for palmar tilt, the difference between observer 2 & 4 measurements plotted against the average measurement for the two observers using data from the first set of measurements

Bland and Altman [25] graphs (Figs. 2 and 3) indicated no clear relationship between an individual measurement and the magnitude of error in measurement. Figures 2 and 3 illustrate this using the example of palmar tilt showing data for observers with high (1 & 5) and low (2&4) measurement correlations.

## Discussion

Distal radius fractures are typically managed non-operatively with cast immobilisation or surgically with either percutaneous pinning (Kirscher wires), external fixation or internal fixation [29]. However, the evidence behind treatment choices based on deformation and radiographic parameters is limited. Best treatment for the various types of distal radius fracture would ideally include a reliable, standardised, evidence-based method of classifying distal radius fractures and unambiguous decision guidelines for treatment.

The rationale for this investigation was to quantify the intra- and inter-observer reliability of eight traditionally reported anatomic parameters in skeletally mature patients with an acute distal radius fracture using PACS computerised images and display systems (Synapse [23]). Bland and Altman [25] graphs (Figs. 2 and 3) indicate no clear relationship between an individual measurement and the magnitude of error in measurement. Consequently, in clinical practice, errors associated with these measurements are better estimated using degrees or millimetres of error (e.g. +/− 4°) than error expressed as a percentage of the range (e.g. 10 % of range).

Despite adopting standardised measurement techniques and observer training, the intra- and inter-observer consistency when applying these measures varied greatly for the eight anatomic parameters. Intra-observer ICC values appeared higher than inter-observer for all anatomic parameters except palmar tilt and may indicate the potential for additional training to remediate inconsistencies between clinicians for measurements.

Kreder et al. [6] published the results of intra- and inter-observer consistency in assessing these eight anatomic parameters with repeated assessments at 0 and 2–4 weeks of six radiographs of healed fractures conducted by 16 observers. Printed films were assessed on flat view boxes and measured using protractors and rulers. Limitations of the Kreder et al. [6] investigation were the small sample of radiographs and that radiographs were of healed fractures.

For intra-observer reliability in the current investigation, ICC values were found to be above 0.80 for palmar tilt and dorsal shift (Table 2). Only one parameter, palmar tilt, was associated with an inter-observer ICC value above 0.80. Unlike the current investigation, Kreder et al. [6] found ulnar variance was the only parameter to have an intra-and inter-observer ICC value above 0.80.

Comparison of error margins in millimeters or degrees (e.g. using the SEM in Table 3) is preferable to comparison of ICC values. This is because the magnitude of ICCs is affected by the range of raw scores included in the computation. This effect is referred to as attenuation of range and has the consequence that the ICC will increase as the variance in raw scores increases despite the same absolute differences (error) in repeated measurements. We were unable to determine the extent to which the higher ICCs obtained in the current investigation were a consequence of a larger range of raw scores as we did not have the variance estimates for Kreder et al.’s [6] data. However, on examination of the range of raw scores for the data analysed in both studies (Table 3), it is possible that attenuation of range might explain at least some of the observed differences.

It is difficult to be unequivocally confident that the use of computerised images and measurement procedures facilitates additional accuracy. If we were studying a similar spectrum of measurements, some differences in study design may account for observed differences. These include the digital methods we employed, our larger number of radiographs (30 versus 6) and that we studied acute fractures while Kreder et al. [6] studied healed fractures. This may have afforded us better visibility of the cortical disruption. The use of acute fracture images that mirror authentic practice confirms Kreder et al.’s [6] findings and extends the validity of claims regarding measurement utility across a representative spectrum of deformity.

Our classification of radiographs into Group 1 (mild deformity) and 2 (severe deformity) was undertaken to enable the spectrum of mild to severe deformity to be represented in the radiographs. While the accuracy of this step cannot be defended based on our analysis of the reliability of radiographic measurements, the range of obtained measurements in this study were comparable to the range of measurements obtained in the study by Kreder et al. [6], suggesting some success in capturing the spectrum of severity.

### Clinical relevance

#### Intra-articular gap and step

A number of investigations have recommended conservative management for distal radius fractures when intra-articular gap or step is less than 1 mm; accelerated development of arthritis, increased severity of degenerative changes and poor functional outcome has been linked with intra-articular gap or step greater than 1 mm [7–9]. Intra-articular gap and step measurements in both the current investigation and previous literature (Tables 2, 4 and 5) were associated with low intra- and inter-reliability ICC values. Given the poor reliability for assessing intra-articular gap or step, we question the suitability of using these radiographic interpretations as criteria for guiding treatment choices. It is possible that additional training in measurement technique might improve the accuracy of these measurements and the cost-benefits of computerised tomography for improving reliability warrants exploration.

#### Dorsal and palmar tilt

It has been argued that functional outcomes are significantly affected when dorsal tilt (negative palmar tilt) exceeds 10° or 12° [10–12] or palmar tilt exceeds 25° [13]. Allowing for error in estimates, dorsal tilt would need to be less than 2.2° to be confident that in 95 % of cases it is actually less than 10°. Error estimates indicate that palmar tilt measurement would need to be less than 17.2° to be confident that in 95 % of cases it is less than 25°.

#### Ulnar variance

Positive ulnar variance greater than 3 mm has been reported to negatively impact functional ability [19, 20]. Allowing for error in estimates we would need to see no more than 0.8 mm of positive ulnar variance to be confident that true ulnar variance is no more than 3 mm.

#### Radial angle

Radial angle generally reduces with displaced distal radius fractures and a radial angle of less than 15° has been used to indicate operative management [14, 15]. We would need to see more than 21.8° radial angle to be confident that in 95 % of cases we have more than 15°.

#### Radial height

A reduction in radial height of 3–6 mm has been linked with a decline in functional outcome [16, 17]. Error estimates and a 95 % CI upper limit of 5.2 mm raise questions about the utility of this measure using the standardised methods described.

#### Dorsal shift and radial shift

There is limited information in the literature linking the measurements of dorsal shift and radial shift with functional outcomes raising questions around the importance of these anatomical parameters for guiding treatment decisions. The current investigation indicates that dorsal shift can be reliably measured [inter-observer ICC value (0.75)] and therefore exploration of its relationship with functional outcomes is warranted.

## Conclusions

In summary, when interpreting computerised images of acute distal radius fractures, reliability measures and error margins from this investigation support the use of palmar tilt, radial angle and ulnar variance measurements for guiding treatment choices. However, consideration needs to be given to error margins when using these measurements to guide treatment choices. Reliability measures and error margins indicate that intra-articular gap and step cannot reliably be used to guide treatment choices for acute distal radius fractures when using the methods for interpreting radiographic parameters investigated in this study. This study did not investigate the reliability of the scan itself, and this warrants further investigation.

The next step from this investigation is to use evidence-based methods to develop decision rules for treatment guidelines following acute distal radius fracture. It is known that clinicians do not routinely measure all eight anatomical parameters in clinical practice. Further investigation is required to quantify whether there is consistency and agreement with the anatomical parameters that clinicians deem important for decision making for acute distal radius fractures.

## Abbreviations

ANOVA, analysis of variance; *BMS*, between-subjects variance; CIs, confidence intervals; ED, Emergency Department; ICCs, intraclass correlation coefficients; MS, mean squares; OCCC, overall concordance correlation coefficient; PA, posteroanterior; PACS, picture archiving and communication system; SEM, standard error of measurement; *WMS*, within subjects variance

## Additional file

Additional file 1: Data set- Repeated measurements by five observers for the eight anatomical parameters. File contains the raw values for the repeated measurements recorded by the five observers for the 30 radiographs. (XLS 49 kb)
